# Fonseca’s Questionnaire Is a Useful Tool for Carrying Out the Initial Evaluation of Temporomandibular Disorders in Dental Students

**DOI:** 10.3390/clinpract14050132

**Published:** 2024-08-26

**Authors:** Valeria Mitro, Angela Rosa Caso, Federica Sacchi, Massimiliano Gilli, Guido Lombardo, Gabriele Monarchi, Stefano Pagano, Antonio Tullio

**Affiliations:** 1 Department of Maxillo-Facial Surgery, Hospital of Perugia, Sant’Andrea Delle Fratte, 06132 Perugia, Italy; valeria.mitro@ospedale.perugia.it (V.M.);; 2 Department of Medicine, Section of Maxillofacial Surgery, University of Siena, Viale Bracci, 53100 Siena, Italygabriele.monarchi@gmail.com (G.M.); 3 Unit of Paediatric Dentistry, Department of Surgical and Biomedical Sciences, University of Perugia, 06132 Perugia, Italy; 4 Department of Medicine and Surgery, Faculty of Dentistry, University of Perugia, Sant Andrea delle Fratte, 06156 Perugia, Italy; 5 Department of Surgical and Biomedical Sciences, Section of Maxillo-Facial Surgery, University of Perugia, Piazzale Gambuli 1, 06129 Perugia, Italy

**Keywords:** Fonseca’s questionnaire, temporomandibular disorders, temporomandibular joint, TMDs

## Abstract

**Background**: Temporomandibular disorders (TMDs) represent a prevalent multifactorial condition that impacts a significant portion of the global population. The objective of this study was to employ Fonseca’s questionnaire for an initial assessment of TMDs. **Methods:** A cross-sectional study was conducted on a sample of 250 undergraduates from the Dental School of the University of Perugia, Italy. The chi-square test, with a significance level set at *p* < 0.05, was used to evaluate a statistically significant relationship between TMDs and several variables such as gender, age, employed/unemployed, and physically active or not. **Results:** The data obtained through the questionnaire indicated that a considerable percentage of students (78%) exhibited signs consistent with TMDs. The most frequently reported signs and symptoms included psychological stress (49.6%), dental clenching and grinding (34%), joint clicking (33.6%), frequent headaches (15.2%), and neck pain (23.2%). Notably, when considering moderate to severe symptoms of TMDs, females were more significantly affected than males. Furthermore, factors such as age, employment status, and physical activity did not appear to influence the prevalence of TMDs. **Conclusions:** The high prevalence of TMDs identified within this young population (university students), as measured by this questionnaire (albeit warranting validation through more rigorous methodologies) underscores the necessity for the implementation of new preventive strategies that specifically address this demographic.

## 1. Introduction

Temporomandibular disorders (TMDs) are among the most prevalent causes of orofacial pain, generally affecting masticatory, pre-auricular, and craniocervicofacial muscles and the temporomandibular joint (TMJ) [1]. According to the Diagnostic Criteria for TMDs (DC/TMD), the gold standard method for diagnosing these disorders, TMDs are a syndrome characterized by joint disc displacement (clicking and/or locking) and muscle disorders (including myofascial pain) with and without mouth opening limitation. Additionally, arthralgia, arthritis, and osteoarthritis can be diagnosed as part of TMDs [2]. Furthermore, there are also some common characteristics that can help identify patients with TMDs: these are generally tense individuals who report frequent headaches and neck pain [3,4]. Finally, the auricular system might be involved in symptoms such as tinnitus, dizziness, otalgia, and a sensation of ear fullness [5]. The chronic pain associated with TMDs could be confused with other types of chronic pain including headaches, fibromyalgia, and other neurological conditions such as allodynia and hyperalgesia [6]. The etiology of TMDs includes multifactorial causes. In the past, altered dental occlusion, due to malocclusions or occlusal interferences, was considered one of the most important etiological factors. More recently, however, its relevance has been debated, with the consequence of limiting occlusal treatment to resolve temporomandibular disorders [7,8,9]. The other risk factors reported in the literature were parafunctions (bruxism and occlusal overload), traumatic dental lesions, psychological diseases, joint hyperlaxity and hypermobility, postural alterations (ascending pathway), muscular hyperfunction, hormonal influences (in particular, estrogens in females), and hereditary factors [8,10,11]. TMDs are the second most common musculoskeletal disorder in the world causing pain and disability [12,13], with an early onset and a tendency to increase over the years, affecting 11% of children and adolescents and 31% of adults [1]. The early and widespread onset of TMDs should prompt oral health specialists (e.g., dentists and maxillofacial physicians) to clinically evaluate this disease as early as possible, in order to prevent complications. An initial easy and rapid screening, suitable for large-scale population evaluation, can be performed by administering specific questionnaires that have been demonstrated to be valid and reliable. Among these questionnaires, the most frequently described in the scientific literature is the one developed by Fonseca [14]. Fonseca’s questionnaire is one of several normally used in dentistry and its related fields such as pediatric dentistry (dental fear), oral surgery (post-surgical pain), cariology (caries-related quality of life), or more generally to understand the degree of oral health-related quality of life [15,16,17,18,19].

In Italy, there is a limited amount of research on the prevalence of TMDs. Among these are two studies conducted on the general population [20,21], while several others were carried out in specific clinical settings (dental clinics and maxillofacial clinics) [22,23,24,25,26,27,28]. In the first two abovementioned studies, the RDCs (Research Diagnostic Criteria) were utilized, which, despite being a standardized system for diagnosing TMDs, are known to be lengthy and complex. Specifically, in the first study conducted by Mobilio et al. in 2011, the prevalence of TMD symptoms was assessed in a sample of Italians, with a particular focus on the correlation of gender and age [20]. In the second study (performed by Paduano et al. in 2020), it was demonstrated that one-third of adolescents exhibited at least one TMD symptom [21]. In other studies, all performed by Manfredini and his co-workers in clinical settings, subjects suffering from TMDs were examined. In these patients, the distribution of signs and symptoms of temporomandibular disorders (e.g., disc displacements, and muscle or joint pain) with their relative risk factors (e.g., bruxism, anxiety, and depression) were examined [22,23,24,25,26,27,28].

The scope of our observational study was to evaluate the prevalence of TMDs using Fonseca’s questionnaire in a young adult population (undergraduate students) attending the Dental School, University of Perugia, Italy.

## 2. Materials and Methods

### 2.1. Study Design

This is an observational, cross-sectional study.

### 2.2. Populations

A total of 250 undergraduates from the Dental School of the University of Perugia, Italy, with an average age of 25 years SD 4.3, 75/250 (30%) males, 47/250 (18.8%) foreigners, and 80/250 (32%) who worked in addition to studying, were selected to respond to Fonseca’s questionnaire. The sample size was established based on a similar previous study utilizing the validation procedures of Fonseca’s questionnaire [28]. In this study, to obtain a power equal to 90% and an α equal to 0.01, the authors established a minimum sample size of 119 participants [29]. All our participants were informed about the study’s purpose and signed a form of informed consent. In addition, this study was approved by the local ethics committee, CER Umbria, Perugia, Italy, under protocol number 456/23, dated 13 September 2023. To allow full availability of the data collected in this study, the entire dataset was reported in Appendix A, Table A1.

### 2.3. Exclusion Criteria

We excluded subjects suffering from chronic inflammatory pathologies resulting from systemic disease involving the temporomandibular joint (such as Rheumatoid Arthritis, Juvenile Idiopathic Arthritis, Spondyloarthropathies, etc.).

### 2.4. Fonseca’s Questionnaire

**Figure 1:**
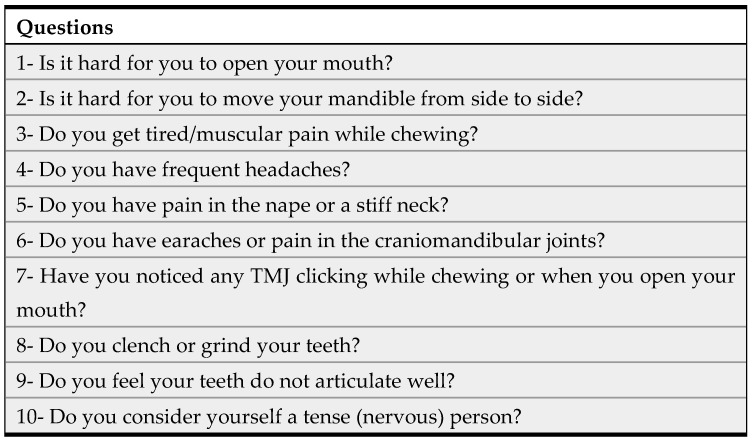
The ten questions included in the English version of Fonseca’s questionnaire.

Fonseca’s questionnaire is a tool for obtaining epidemiological data about TMD prevalence and severity (Figure 1). It consists of 10 questions, 5 evaluating the emotional state of the patient and their perception of TMDs, and the other 5 examining clinical parameters such as pain in the temporomandibular joint, head, or back, or while chewing, as well as parafunctional habits, movement limitation, and joint clicking. For each question, the possible answers were “yes” (10 points), “no” (0 points), and “sometimes” (5 points), with a total score ranging from 0 to 100. Questionnaire scores ranging from 16 to 44 TMDs are classified as mild, from 45 to 69 as moderate, and for higher scores as severe, while for scores lower than 16, TMDs are considered absent.

Before starting this study, Fonseca’s questionnaire underwent translation from its original Portuguese version to an Italian one. This translation process involved two expert translators proficient in both Italian and Portuguese, who initially worked independently, resulting in two distinct translations. Subsequently, a single version was derived through a collaborative assessment by the two translators and the researchers involved in this study. Finally, the Italian translation was back-translated into Portuguese and compared with the original version by the researchers to ensure the maintenance of substantial conceptual equivalence. At this stage, the questionnaire was administered to a part of the initial sample of participants (the 3rd-year students) to assess its comprehensibility, and any suggestions regarding understanding the questions were put into action.

### 2.5. Data Collection

All undergraduate students (n = 250) of the Dental School of the University of Perugia, Italy, were asked to participate in the present study. The questionnaires were administered in September 2023 to the students who gave their informed consent in the Dental School classrooms. After the questionnaires were completed, they were collected in an appropriate container without any signature to ensure anonymity.

### 2.6. Statistical Analysis

The collected data are presented in the percentage frequency tables (Table 1, Table 2, Table 3 and Table 4), which include gender, age, whether they were employed, and whether they were physically active, with the distribution of participants classified according to TMD severity. For qualitative data, the statistical analysis used was the Chi-square test. The level of significance was set at *p* < 0.05. Processing was performed with SPSS, version 25 software for Windows (IBM corp, Armonk, NY, USA).

## 3. Results

All 250 students (175 females) participated in this epidemiological study, with no attrition bias. Fonseca’s questionnaire allowed for the classification of 250 participants as follows: 55/250 (22%) TMD free (with healthy TMJ), while the remaining participants (n = 195/250; 78%) were found to be affected by TMDs. Of these, 136/250 (54.4%) showed a mild degree of the disease, 51/250 (20.4%) moderate, and 8/250 (3.2%) severe. Significant gender differences were found in the prevalence of participants with moderate and severe TMDs, with females more affected than males (28.6% (n = 50/175) vs. 12% (n = 9/75), *p*-value < 0.01), as shown in Table 1.

The results considering age instead (19–23 vs. 23–27 vs. 27–30 years) are illustrated in Table 2.

The most frequently reported signs and symptoms were the following: psychological stress (49.6%, n = 124/250), teeth clenching and grinding (34%, n = 85/250), temporomandibular clicking (33.6%, n = 84/250), frequent headache (15.2%, n = 38/250), and neck pain (23.2%, n = 58/250) (Table 3 and Table 4).

## 4. Discussion

Fonseca’s questionnaire is a useful tool for evaluating the prevalence and severity of TMDs in a young population such as university students due to its diagnostic advantages: low cost, short use times, easy administration, and validity–reliability in its diagnosis [29,30,31,32,33,34,35,36]. The data collected through this questionnaire evidenced that, in the population of Italian undergraduate dental students of Perugia University, three out of four participants (78%) experienced at least one sign or symptom of TMDs. These epidemiologic data were significantly higher than those reported in a recent systematic review of the literature, attesting that the overall prevalence of TMDs was approximately 31% for adults/the elderly and 11% for children/adolescents [1]. To some extent, this difference in terms of prevalence could be attributed to the different methods used for evaluation in the two studies. Specifically, in the systematic review (with its included studies), the TMD diagnosis was carried out using the traditional method of the Research Diagnostic Criteria (RDCs), while in our study, Fonseca’s questionnaire was used. Another explanation could be due to a greater attention by the Dental School students to TMD semiology (as part of their field of scientific interests) as opposed to the general population. A third explanation could be related to incidental conditions that involved all the students, such as the fact that the questionnaire was administered immediately following a stressful exam period. In any case, Fonseca’s questionnaire must be adopted by clinicians only for an initial screening of TMDs before subjecting patients to a detailed evaluation according to the Research Diagnostic Criteria (RDCs). In other words, Fonseca’s questionnaire, due to its easy and rapid adoption, can serve to identify subjects at risk of TMDs. Therefore, although during the specialist visit, some patients indicated as suffering from TMDs by the questionnaire were found to be “false positives”, this eventuality does not alter the clinical usefulness of using the questionnaire [33,37,38,39]. Fonseca recommended a specialist visit only for patients with questionnaire scores indicating moderate or severe TMDs [40]. However, in our approach, we advocate consultation even for those with mild symptoms. This approach is rooted in the belief that even though individuals with mild symptoms may not necessarily require traditional treatment (e.g., occlusal appliances), they can benefit from counseling therapy due to a biopsychosocial model of this disease [41]. Such knowledge equips them with the ability to understand the TMDs’ potential progression, alleviating unnecessary concerns and enabling the adoption of preventative measures and precautions to avert further medical issues.

Another interesting result found in the present study was the lack of a relationship between both employment status and engagement in regular physical activities. As for employment status, a possible explanation could be linked to the probable low number of working hours (for example, only in the evenings) due to their daytime usually required for studying and other university activities. In other words, it is difficult to have a full-time job while attending college. In the literature, a weak relationship between socioeconomic status and temporomandibular disorder was highlighted, but a direct relationship with employment was not found [42]. Similarly, regarding the people engaged in regular physical activity, which reduces stress [43,44] and improves body posture (both related to TMDs) [45], the specific type of activity carried out by participants in this study, whether it be sporting (with daily training) or only occasionally as a hobby, should be clearly defined.

### 4.1. Study Limitations

Although Fonseca’s questionnaire has been validated in several countries around the world (e.g., Brazil, Peru, China, Malaysia, Spain, and Turkey), it has not yet been validated in the Italian version used in our study [29,32,33,34,35,36]. The high prevalence values that emerged from this study raise doubt as to the accuracy of the questionnaire, which could overestimate the prevalence of TMDs. Another limitation of this study could be that we chose as participants only dental students (homogenous group), resulting in an indirectness bias (see the GRADE methodological quality assessment) in relation to the general population. The selection of the sample from only one setting contributed to these limitations.

### 4.2. Clinical Relevance of This Study

The characteristics of the questionnaire (specifically, its ease and speed of use) along with its acceptable level of precision, as demonstrated by previous validation studies [29,32,33,34,35,36], facilitate initial screening for temporomandibular disorders (TMDs) by health professionals outside of, but related to, dental practice, including speech-language therapists, general practitioners, pediatricians, dental hygienists, and nurses [46,47]. This multidisciplinary approach has the potential to broaden the scope of TMD screenings and increase the number of participants eligible for testing. Of course, the administration of the questionnaire cannot take place without a healthcare professional capable of explaining the meaning of words belonging to the medical field which might not be understandable to the general population to whom the questionnaire is addressed. The importance of an early diagnosis (with all the tools available) is linked to the possibility of carrying out an effective treatment against these disorders through a series of non-invasive therapies that include educational interventions, psychological therapies (i.e., cognitive behavior therapy), pharmacological therapies (nonsteroidal anti-inflammatory drugs, muscle relaxants, benzodiazepines, and antidepressants), physical therapy, and occlusal devices [48].

## 5. Conclusions

The high prevalence of TMDs found in Dental School undergraduates should stimulate health policies focused on early disease detection and control (e.g., starting in adolescents) to prevent underestimation and/or delay in diagnosis, which could result in increased severity. In this regard, primary care dentists could play an important role. To obtain more accurate data on the prevalence and severity of TMDs, further methodologically well-conducted studies with larger participants samples from various different settings are needed.

## Figures and Tables

**Table 1:** Gender distribution of participants evaluated and classified according to TMD severity.

TMD Severity	F	M	Total
Absent/Mild	71.43%	88.00%	76.40%
Moderate/Severe	28.57%	12.00%	23.60%
Total	100.00%	100.00%	100.00%

**Table 2:** Age distribution of participants evaluated and classified according to TMD severity.

Age of Participants	Absent/Mild	Moderate/Severe	Total
20–23	37.17%	32.20%	36.00%
24–26	49.21%	50.85%	49.60%
27–30	13.61%	16.95%	14.40%
Total	100.00%	100.00%	100.00%

**Table 3:** Distribution of participants (employed vs. unemployed) according to TMD severity.

TMD Severity	Employed	Unemployed	Total
Absent	15	40	55
Mild	47	89	136
Moderate	15	36	51
Severe	3	5	8
Total	80	170	250

**Table 4:** Distribution of participants (engaged in regular physical activity vs. not engaged) according to TMD severity.

Regular Physical Activity	Absent/Mild	Moderate/Severe	Total
No	36.65%	38.98%	37.20%
Yes	63.35%	61.02%	62.80%
Total	100.00%	100.00%	100.00%

## Data Availability

The data presented in this study are available on request from the corresponding author due to (specify the reason for the restriction).

## Appendix A

**Table A1:** The table shows the complete data set of the study which includes the personal data of the participants, some of their habits and their answers to the ten questions (items) of the Fonseca questionnaire with the related scores.

Name	Gender (M/F)	Age (Years)	Answers to Fonseca’s Questionnaire for Each of the 10 Questions (Items)										Fonseca’s Questionnaire Total Score	Severity Level of TMDs	TMDs Presence (or Not)
			Item 1	Item 2	Item 3	Item 4	Item 5	6 item	Item 7	Item 8	Item 9	Item 10			
M.N	F	24–26	No	No	Sometimes	Yes	Sometimes	Sometimes	Yes	No	No	Sometimes	40	Mild	No
A.P	F	20–23	Sometimes	Sometimes	Sometimes	No	Sometimes	Yes	Yes	No	Yes	Yes	60	Moderate	Yes
G.N	F	24–26	No	No	No	No	No	No	No	No	Sometimes	Yes	15	Not present	No
K.A	F	24–26	No	No	Yes	No	Yes	No	No	Yes	No	Yes	50	Moderate	Yes
F.B	M	27–30	No	No	No	Yes	Yes	No	No	Yes	No	Yes	50	Moderate	Yes
R.L	M	20–23	No	No	No	No	No	No	Sometimes	Yes	No	Sometimes	20	Mild	No
F.F	F	24–26	Sometimes	Yes	Yes	No	No	Yes	Yes	No	No	Yes	70	Severe	Yes
R.T	M	24–26	No	No	Sometimes	No	No	No	No	Sometimes	Sometimes	Yes	20	Mild	No
A.F	F	20–23	Sometimes	Sometimes	Sometimes	Yes	Yes	Yes	No	Sometimes	No	Sometimes	55	Moderate	Yes
A.L	F	20–23	No	No	No	Yes	Yes	No	Sometimes	Yes	No	Sometimes	40	Mild	No
M.F	F	20–23	No	No	No	No	No	No	No	No	No	Sometimes	5	Not present	No
S.B	F	27–30	No	Sometimes	Sometimes	Yes	Yes	Sometimes	No	Yes	No	Yes	55	Moderate	Yes
S.W	M	24–26	No	No	Sometimes	No	No	No	No	Sometimes	No	Yes	20	Mild	No
L.S	F	24–26	No	Sometimes	No	Sometimes	No	No	Sometimes	No	Yes	Yes	35	Mild	No
M.T	F	24–26	No	Sometimes	No	Sometimes	Sometimes	Yes	Yes	Sometimes	Yes	No	50	Moderate	Yes
G.P	F	24–26	No	No	No	No	Sometimes	No	Yes	Sometimes	No	No	20	Mild	No
F.R	F	24–26	Sometimes	No	No	Sometimes	No	No	Yes	Sometimes	No	Yes	35	Mild	No
V.P	M	24–26	No	No	No	No	No	No	No	Yes	No	No	10	Not present	No
L.G	F	20–23	No	No	No	Sometimes	Sometimes	No	Sometimes	Yes	No	Yes	35	Mild	No
A.G	F	24–26	No	No	No	No	No	No	Sometimes	Sometimes	No	No	10	Not present	No
G.M	F	20–23	Sometimes	No	Sometimes	No	No	Sometimes	Sometimes	No	Sometimes	Yes	35	Mild	No
F.DP	M	27–30	No	No	No	No	No	No	No	No	No	Sometimes	5	Not present	No
M.T	M	20–23	No	No	Sometimes	Sometimes	Yes	No	No	Yes	No	Sometimes	35	Mild	No
F.A	F	24–26	No	No	No	Sometimes	Sometimes	Sometimes	Yes	Yes	No	Sometimes	40	Mild	No
P.L	M	24–26	No	No	No	Sometimes	Yes	No	No	No	No	Sometimes	20	Mild	No
N.P	F	24–26	Sometimes	Sometimes	Sometimes	No	Sometimes	No	Yes	No	No	Yes	40	Mild	No
N.PL	F	24–26	No	No	No	Sometimes	Yes	No	No	No	Yes	Yes	35	Mild	No
M.T	F	27–30	No	No	No	Sometimes	Sometimes	No	No	No	Yes	Yes	30	Mild	No
E.N	M	24–26	No	No	No	No	Sometimes	No	No	No	No	Sometimes	10	Not present	No
F.G	F	27–30	No	No	No	No	No	No	No	No	No	Yes	10	Not present	No
A.B	F	20–23	No	No	No	No	No	No	Yes	No	No	Sometimes	15	Not present	No
F.P	M	27–30	No	No	No	No	No	No	Yes	Sometimes	Yes	Yes	35	Mild	No
P.P	F	24–26	No	No	No	No	Sometimes	No	No	No	No	Sometimes	10	Not present	No
E.L	F	24–26	No	No	No	Sometimes	Sometimes	Yes	Yes	Yes	No	Sometimes	45	Moderate	Yes
M.L	F	24–26	Sometimes	Yes	Yes	No	Yes	Sometimes	Yes	Yes	Yes	Yes	75	Severe	Yes
A.O	F	27–30	No	Sometimes	Sometimes	Sometimes	Yes	Sometimes	Yes	No	Yes	Yes	60	Moderate	Yes
M.M	F	24–26	No	Sometimes	Sometimes	No	Sometimes	Sometimes	Yes	Yes	Yes	Sometimes	55	Moderate	Yes
A.B	F	20–23	Sometimes	No	No	Sometimes	Yes	Sometimes	Yes	Yes	No	Yes	55	Moderate	Yes
G.A	F	20–23	No	No	Sometimes	Sometimes	Yes	No	Sometimes	Yes	Sometimes	Sometimes	45	Moderate	Yes
T.M	F	24–26	No	Sometimes	No	Sometimes	Yes	Sometimes	Yes	Sometimes	No	Sometimes	45	Moderate	Yes
M.M	F	24–26	No	No	Sometimes	Yes	Yes	Sometimes	Sometimes	Sometimes	No	Yes	50	Moderate	Yes
D.GP	F	24–26	No	No	Sometimes	No	No	No	Yes	Sometimes	Yes	No	30	Mild	No
M.D	M	24–26	No	No	No	No	Yes	No	No	Sometimes	No	Yes	25	Mild	No
S.C	F	24–26	No	No	Sometimes	Yes	Yes	Yes	No	Yes	Sometimes	Yes	60	Moderate	Yes
A.P	F	20–23	No	No	Sometimes	No	No	No	No	Sometimes	No	Yes	20	Mild	No
V.O	F	20–23	No	No	No	Yes	Sometimes	No	No	Sometimes	No	Sometimes	25	Mild	No
M.F	F	20–23	No	No	Sometimes	No	No	No	No	Yes	No	Sometimes	20	Mild	No
C.F	F	20–23	No	No	Yes	No	Yes	No	No	No	Yes	Yes	40	Mild	No
M.M	M	20–23	No	No	Sometimes	No	No	No	Sometimes	Yes	Yes	Yes	40	Mild	No
R.F	F	20–23	No	No	No	Yes	Yes	Yes	Yes	Sometimes	No	Yes	55	Moderate	Yes
A.A	M	27–30	No	No	No	No	No	No	No	No	No	No	0	Not present	No
F.M	F	24–26	Sometimes	Sometimes	Sometimes	No	No	Yes	Yes	No	Yes	Yes	55	Moderate	Yes
S.C	F	20–23	Sometimes	No	Yes	Sometimes	No	Yes	Yes	Yes	No	Yes	55	Moderate	Yes
S.R	F	24–26	No	No	No	No	No	No	Yes	No	No	Sometimes	15	Not present	No
M.P	F	24–26	No	No	No	Sometimes	No	No	Yes	Yes	Yes	Yes	45	Moderate	Yes
M.R	F	24–26	No	No	Sometimes	Sometimes	Sometimes	Yes	No	Yes	No	Yes	45	Moderate	Yes
S.A	M	24–26	No	No	No	Yes	Yes	No	No	No	No	Yes	30	Mild	No
E.P	F	20–23	No	No	No	No	No	No	No	Sometimes	No	Yes	15	Not present	No
M.B	F	20–23	No	No	Sometimes	No	Sometimes	No	Sometimes	Sometimes	Yes	Yes	40	Mild	No
M.E	F	27–30	No	No	No	Sometimes	Yes	No	No	Yes	No	Yes	35	Mild	No
G.V	F	20–23	No	No	No	No	No	No	No	Yes	No	Sometimes	15	Not present	No
S.B	F	24–26	No	No	No	Yes	Sometimes	No	Sometimes	Sometimes	Sometimes	Yes	40	Mild	No
G.Z	F	20–23	No	No	No	No	No	No	Sometimes	Sometimes	Yes	Sometimes	25	Mild	No
F.T	F	24–26	Sometimes	Sometimes	Sometimes	Yes	No	No	Yes	Yes	No	Yes	55	Moderate	Yes
V.V	F	24–26	No	No	No	Sometimes	Sometimes	No	No	No	No	Sometimes	15	Not present	No
E.V	F	20–23	No	No	No	No	No	No	No	No	No	Yes	5	Not present	No
G.C	F	24–26	No	Yes	Sometimes	Sometimes	No	Sometimes	Sometimes	Yes	Sometimes	Yes	55	Moderate	Yes
S.C	F	24–26	No	No	No	No	No	No	Yes	Yes	No	Yes	30	Mild	No
M.AM	M	24–26	No	No	No	Yes	Yes	No	Sometimes	Sometimes	No	Sometimes	35	Mild	No
B.A	F	24–26	No	No	No	Sometimes	No	No	Sometimes	Yes	Yes	Yes	40	Mild	No
L.G	F	24–26	No	No	No	Sometimes	No	No	Yes	Sometimes	No	Sometimes	25	Mild	No
S.B	F	20–23	No	No	No	No	No	No	No	No	No	Sometimes	5	Not present	No
T.C	F	24–26	No	No	No	No	No	No	Sometimes	Sometimes	No	Yes	20	Mild	No
D.V	M	24–26	No	No	No	No	No	No	No	Sometimes	Sometimes	Yes	20	Mild	No
D.T	F	24–26	No	No	No	No	No	Sometimes	Yes	No	No	Sometimes	20	Mild	No
E.R	F	20–23	No	No	No	No	No	No	Sometimes	No	No	Sometimes	10	Not present	No
P.P	F	27–30	No	Yes	No	No	No	No	No	Yes	No	Sometimes	25	Mild	No
V.A	F	24–26	No	Sometimes	Sometimes	Sometimes	Yes	Sometimes	Sometimes	Sometimes	No	Yes	50	Moderate	Yes
P.B	F	27–30	No	No	No	No	No	No	No	Yes	Sometimes	Sometimes	20	Mild	No
L.B	M	24–26	No	No	Sometimes	No	Sometimes	No	Sometimes	No	No	Sometimes	20	Mild	No
E.N	F	27–30	Sometimes	No	No	No	No	No	Yes	Sometimes	Yes	No	30	Mild	No
G.P	F	24–26	No	Yes	Sometimes	Sometimes	No	No	Yes	Yes	Yes	Yes	55	Moderate	Yes
C.N	M	20–23	No	No	No	No	Yes	No	No	Yes	No	Yes	30	Mild	No
L.C	M	24–26	No	Yes	Yes	Sometimes	Sometimes	Sometimes	No	Yes	Yes	Yes	65	Moderate	Yes
M.L	F	24–26	No	No	No	No	Sometimes	Sometimes	Sometimes	Sometimes	Yes	Sometimes	35	Mild	No
P.V	F	24–26	No	No	No	No	No	No	Yes	Yes	No	Yes	30	Mild	No
R.M	M	27–30	No	No	No	No	No	No	No	Sometimes	No	Sometimes	10	Not present	No
D.B	F	20–23	No	No	Yes	No	No	No	Yes	Yes	Yes	Yes	50	Moderate	Yes
S.L	F	24–26	Sometimes	No	No	Sometimes	Sometimes	No	Yes	Yes	Yes	Yes	55	Moderate	Yes
C.C	F	20–23	Sometimes	No	No	No	No	No	Sometimes	No	No	Sometimes	15	Not present	No
G.R	M	20–23	No	No	No	No	No	No	Sometimes	No	No	No	5	Not present	No
F.R	F	20–23	No	No	No	Sometimes	Sometimes	No	No	Sometimes	No	Sometimes	20	Mild	No
C.C	M	24–26	No	No	Sometimes	Sometimes	Sometimes	No	Sometimes	No	No	Sometimes	25	Mild	No
M.T	F	20–23	Sometimes	No	No	Yes	Sometimes	No	Sometimes	No	Yes	No	35	Mild	No
F.M	F	24–26	Sometimes	No	Sometimes	Sometimes	Yes	No	No	Yes	Yes	Yes	55	Moderate	Yes
S.C	F	24–26	No	No	No	Sometimes	Sometimes	No	Sometimes	Sometimes	Yes	Yes	40	Mild	No
D.T	F	24–26	No	No	No	No	No	No	No	No	No	Sometimes	5	Not present	No
G.L	F	24–26	No	No	No	No	No	No	Yes	No	Sometimes	Sometimes	20	Mild	No
L.T	F	24–26	Sometimes	Sometimes	Sometimes	Yes	Yes	Yes	Yes	Yes	Yes	Yes	85	Severe	Yes
A.P	M	20–23	No	No	No	No	No	No	No	Sometimes	Yes	Sometimes	20	Mild	No
VN.T	F	24–26	No	No	No	No	No	No	No	No	Yes	Yes	20	Mild	No
F.O	F	24–26	No	No	Sometimes	Sometimes	Yes	No	Sometimes	Sometimes	No	Yes	40	Mild	No
A.MR	M	24–26	No	No	Sometimes	No	No	No	Sometimes	No	Yes	Sometimes	25	Mild	No
S.B	M	27–30	No	No	No	No	No	No	No	Yes	No	Yes	20	Mild	No
A.T	M	24–26	No	No	No	No	No	No	No	Yes	No	Yes	20	Mild	No
A.M	M	24–26	No	No	No	No	No	Sometimes	No	Yes	Sometimes	Sometimes	25	Mild	No
S.L	M	27–30	No	No	No	No	No	No	Yes	Sometimes	Sometimes	Sometimes	25	Mild	No
R.D	M	24–26	No	No	No	No	Sometimes	No	No	No	Sometimes	Yes	20	Mild	No
T.A	F	24–26	No	No	No	Yes	Sometimes	No	No	No	No	Sometimes	20	Mild	No
M.P	F	20–23	No	No	No	Sometimes	Sometimes	No	No	No	Yes	Sometimes	25	Mild	No
P.G	F	24–26	Sometimes	No	Sometimes	No	No	No	Yes	Sometimes	Yes	No	35	Mild	No
M.V	F	24–26	No	No	Yes	No	No	No	Yes	No	No	Yes	30	Mild	No
A.A	F	24–26	No	No	No	No	No	No	No	Sometimes	Sometimes	Sometimes	15	Not present	No
B.L	M	20–23	No	No	Sometimes	No	Sometimes	No	Yes	No	No	No	20	Mild	No
G.G	M	20–23	No	No	No	No	No	No	Yes	Yes	Yes	Yes	40	Mild	No
N.F	M	27–30	No	No	Sometimes	Yes	Yes	Yes	Yes	Yes	Yes	Yes	75	Severe	Yes
A.B	F	20–23	No	No	No	Sometimes	Yes	No	No	No	No	Yes	25	Mild	No
F.D	F	24–26	Yes	Yes	No	Sometimes	Yes	Sometimes	Yes	Sometimes	Yes	Sometimes	70	Severe	Yes
F.G	F	24–26	No	No	Sometimes	Yes	Yes	Sometimes	No	Yes	Yes	Yes	60	Moderate	Yes
A.R	F	20–23	Sometimes	No	No	No	No	No	Yes	No	No	Sometimes	20	Mild	No
I.F	F	20–23	Yes	Sometimes	Sometimes	Yes	Sometimes	Sometimes	Yes	Yes	Yes	Yes	80	Severe	Yes
O.B	F	27–30	Sometimes	Sometimes	Yes	Sometimes	Yes	No	Yes	Yes	No	Yes	65	Moderate	Yes
M.C	M	24–26	No	Sometimes	No	No	No	Yes	Yes	No	Yes	Yes	45	Moderate	Yes
O.D	M	24–26	No	No	No	No	No	No	Sometimes	No	No	No	5	Not present	No
P.A	M	24–26	No	No	No	No	No	No	No	Yes	No	Yes	20	Mild	No
R.A	F	24–26	No	No	Sometimes	No	No	No	Sometimes	Yes	Sometimes	Yes	35	Mild	No
B.G	F	24–26	Yes	Sometimes	Yes	Yes	Sometimes	No	Yes	Yes	Sometimes	Yes	75	Severe	Yes
M.B	F	20–23	No	No	No	No	Yes	No	Yes	Yes	No	Yes	40	Mild	No
G.R	F	24–26	Sometimes	No	No	Sometimes	No	No	No	Yes	No	Yes	30	Mild	No
A.L	F	20–23	No	No	Sometimes	No	No	No	No	Yes	Sometimes	Sometimes	25	Mild	No
MG.D	F	20–23	No	No	No	No	No	No	No	Sometimes	No	Sometimes	10	Not present	No
S.B	M	20–23	No	No	No	No	No	No	No	No	No	No	0	Not present	No
J.P	F	24–26	No	No	No	Sometimes	No	No	No	Sometimes	No	Yes	20	Mild	No
MK.L	F	20–23	No	No	No	No	No	No	Sometimes	Sometimes	No	Yes	20	Mild	No
A.A	F	24–26	Sometimes	No	Sometimes	No	No	No	Sometimes	No	Yes	Sometimes	30	Mild	No
F.C	F	24–26	Sometimes	No	Sometimes	Sometimes	Sometimes	No	Yes	Sometimes	No	Sometimes	40	Mild	No
V.N	F	24–26	No	No	No	No	Yes	No	Sometimes	Yes	No	Yes	35	Mild	No
L.V	M	20–23	No	No	No	No	No	No	Yes	No	No	No	10	Not present	No
S.B	F	24–26	No	No	No	No	Yes	No	Sometimes	Sometimes	Sometimes	No	25	Mild	No
A.DL	M	20–23	No	No	No	Yes	Yes	No	Sometimes	No	No	Yes	35	Mild	No
G.A	M	20–23	Sometimes	No	No	No	No	No	Yes	Yes	Yes	Sometimes	40	Mild	No
R.B	M	20–23	No	No	No	Yes	Yes	No	No	Yes	No	Yes	40	Mild	No
A.B	F	24–26	No	No	Sometimes	No	Sometimes	Sometimes	Sometimes	Sometimes	Sometimes	Yes	40	Mild	No
D.Z	M	20–23	No	No	No	No	No	No	No	No	No	No	0	Not present	No
C.G	F	24–26	No	No	No	No	Yes	No	No	No	Yes	Sometimes	25	Mild	No
M.T	F	24–26	No	No	No	Yes	No	No	Yes	No	Yes	Yes	40	Mild	No
R.C	F	20–23	Sometimes	Sometimes	Sometimes	Sometimes	No	Sometimes	Sometimes	No	No	Sometimes	35	Mild	No
G.O	M	27–30	No	No	No	Yes	Yes	No	No	No	No	Sometimes	25	Mild	No
B.C	F	20–23	No	No	No	No	Sometimes	No	No	No	Sometimes	Yes	15	Not present	No
P.R	M	24–26	No	No	No	No	No	No	No	Sometimes	No	No	5	Not present	No
F.G	M	24–26	No	No	No	Sometimes	No	No	No	Yes	Yes	No	25	Mild	No
G.S	F	24–26	No	No	No	No	No	No	Yes	Sometimes	No	Sometimes	20	Mild	No
F.B	F	27–30	No	No	Sometimes	Sometimes	Sometimes	Yes	No	No	No	Yes	35	Mild	No
M.L	F	20–23	No	No	No	No	No	No	No	No	No	Sometimes	5	Not present	No
S.W	M	20–23	No	No	No	No	No	No	Yes	Yes	No	No	20	Mild	No
S.S	F	20–23	Sometimes	No	Yes	No	Yes	No	Yes	Yes	No	Yes	55	Moderate	Yes
P.B	M	24–26	No	No	No	No	No	No	Yes	No	No	No	10	Not present	No
AM.A	F	24–26	No	No	No	No	Sometimes	No	Sometimes	Sometimes	No	No	15	Not present	No
S.B	F	20–23	No	No	No	No	Sometimes	No	Yes	Yes	Yes	Sometimes	40	Mild	No
B.L	M	27–30	Sometimes	No	Yes	No	Sometimes	No	Yes	Yes	No	Sometimes	45	Moderate	Yes
R.V	F	24–26	Sometimes	No	Sometimes	Sometimes	No	Sometimes	Sometimes	No	No	Sometimes	30	Mild	No
C.B	F	27–30	No	No	Sometimes	Yes	Sometimes	No	No	Yes	No	Yes	40	Mild	No
A.B	F	20–23	No	No	Sometimes	No	No	No	Yes	Yes	No	Yes	35	Mild	No
P.C	F	20–23	No	No	Sometimes	Sometimes	Sometimes	Sometimes	No	Sometimes	Yes	Yes	45	Moderate	Yes
P.B	F	27–30	No	No	Yes	No	No	Sometimes	Yes	Yes	Yes	Yes	50	Moderate	Yes
A.A	F	24–26	No	No	No	No	No	No	No	No	No	Sometimes	5	Not present	No
A.B	F	24–26	No	No	No	No	No	No	No	Yes	No	No	10	Not present	No
L.S	F	20–23	No	No	No	No	No	No	No	No	No	No	0	Not present	No
L.C	F	24–26	No	No	No	No	No	No	No	No	No	Yes	10	Not present	No
P.B	F	24–26	No	Yes	Sometimes	Yes	Yes	No	No	Yes	Yes	Yes	65	Moderate	Yes
E.R	F	20–23	No	No	No	Yes	Yes	Yes	No	No	Yes	Yes	50	Moderate	Yes
L.B	F	24–26	Sometimes	No	No	No	Yes	Yes	Sometimes	Yes	No	Sometimes	40	Mild	No
V.A	M	27–30	Sometimes	No	Yes	Sometimes	No	No	No	Yes	Yes	Sometimes	45	Moderate	Yes
F.B	F	27–30	Sometimes	Sometimes	Yes	Yes	No	No	No	Yes	No	Yes	50	Moderate	Yes
F.F	M	24–26	No	No	No	No	Yes	No	No	No	Yes	Sometimes	20	Mild	No
A.M	F	24–26	No	No	No	Sometimes	No	No	Sometimes	Sometimes	No	Sometimes	20	Mild	No
P.V	F	20–23	No	No	No	Yes	Yes	No	No	No	No	Yes	30	Mild	No
M.V	F	24–26	No	No	Sometimes	No	No	No	Sometimes	No	No	Yes	20	Mild	No
P.S	F	24–26	No	No	No	No	No	No	No	No	No	No	0	Not present	No
N.S	F	24–26	No	No	No	No	No	No	Yes	Sometimes	No	No	15	Not present	No
L.R	F	27–30	No	No	No	No	Sometimes	No	Sometimes	No	No	Sometimes	15	Not present	No
E.Z	F	24–26	No	No	No	No	Yes	No	No	Yes	Sometimes	Yes	35	Mild	No
F.R	F	24–26	No	No	No	No	Sometimes	No	No	No	Yes	No	15	Not present	No
S.B	F	27–30	No	No	No	No	Sometimes	No	No	Sometimes	Yes	Sometimes	25	Mild	No
B.E	F	27–30	Sometimes	No	No	No	No	No	Yes	No	Yes	Yes	35	Mild	No
B.G	M	24–26	No	No	No	Sometimes	No	No	Yes	Yes	No	Yes	35	Mild	No
T.F	M	27–30	No	Yes	No	Sometimes	Yes	No	Yes	No	No	Sometimes	40	Mild	No
AR.G	F	20–23	No	No	No	No	No	No	Sometimes	No	No	No	5	Not present	No
M.B	M	27–30	No	No	Sometimes	No	No	No	Sometimes	Sometimes	Sometimes	Sometimes	25	Mild	No
A.F	F	24–26	No	No	No	No	No	No	No	No	No	Sometimes	5	Not present	No
D.H	F	20–23	Sometimes	No	No	Yes	Yes	Yes	No	Yes	Yes	Yes	65	Moderate	Yes
E.P	M	24–26	No	No	Sometimes	Sometimes	No	No	Sometimes	Sometimes	No	Yes	30	Mild	No
F.P	F	20–23	No	No	No	No	No	No	Yes	No	Yes	Yes	30	Mild	No
M.L	F	24–26	No	No	No	No	No	No	Sometimes	No	No	No	5	Not present	No
S.C	F	24–26	No	No	No	No	No	No	No	No	No	Sometimes	5	Not present	No
R.B	F	20–23	No	No	No	No	Yes	No	No	No	No	Yes	20	Mild	No
A.P	M	24–26	Sometimes	Sometimes	No	Sometimes	No	No	No	Yes	Yes	Sometimes	35	Mild	No
S.A	M	24–26	No	No	No	Yes	Yes	No	No	No	Yes	Yes	40	Mild	No
E.R	M	24–26	No	No	No	No	No	No	No	Yes	No	No	10	Not present	No
F.P	M	24–26	No	Yes	Sometimes	Sometimes	Sometimes	Sometimes	No	Sometimes	Yes	Yes	55	Moderate	Yes
M.M	F	27–30	No	Sometimes	Sometimes	Sometimes	Sometimes	Sometimes	No	Sometimes	Sometimes	Sometimes	40	Mild	No
C.M	M	24–26	No	No	No	No	No	No	No	Yes	No	No	10	Not present	No
A.B	M	24–26	No	No	No	No	Sometimes	Sometimes	Yes	No	No	Yes	30	Mild	No
G.I	M	24–26	No	No	No	No	No	No	No	Sometimes	Sometimes	Sometimes	15	Not present	No
S.P	F	20–23	No	No	No	No	No	No	No	Yes	Yes	No	20	Mild	No
E.P	F	20–23	No	No	No	No	No	No	No	Yes	No	Yes	20	Mild	No
I.U	F	24–26	No	No	Sometimes	No	Sometimes	No	No	No	Yes	Yes	30	Mild	No
D.A	F	20–23	No	No	No	No	No	No	Yes	No	Yes	Yes	30	Mild	No
P.A	M	20–23	No	No	Sometimes	No	No	Sometimes	Yes	Yes	Yes	Sometimes	45	Moderate	Yes
S.S	M	24–26	No	No	Yes	No	No	No	Sometimes	No	Yes	Sometimes	30	Mild	No
N.M	F	20–23	No	No	Sometimes	No	Sometimes	Sometimes	Yes	Yes	Yes	No	45	Moderate	Yes
D.V	F	20–23	No	No	Sometimes	No	Yes	No	No	Sometimes	Yes	Yes	40	Mild	No
S.B	M	20–23	No	No	No	Sometimes	Sometimes	Sometimes	Sometimes	Yes	Yes	Yes	50	Moderate	Yes
O.D	F	24–26	No	No	Sometimes	Sometimes	Sometimes	Sometimes	Sometimes	Sometimes	Sometimes	Yes	45	Moderate	Yes
N.V	M	20–23	No	No	No	Sometimes	Yes	No	No	No	No	Sometimes	20	Mild	No
R.M	M	24–26	No	No	No	Sometimes	No	No	Sometimes	No	No	Sometimes	15	Mild	No
M.F	M	20–23	No	No	No	Yes	No	No	Yes	Sometimes	No	Yes	35	Mild	No
G.M	F	20–23	No	Sometimes	Sometimes	No	No	No	Sometimes	No	Yes	Yes	35	Mild	No
A.R	F	27–30	No	No	Sometimes	Yes	Yes	Sometimes	Yes	Sometimes	Yes	Sometimes	55	Moderate	Yes
F.R	M	27–30	No	No	No	No	No	No	Yes	No	No	No	10	Not present	No
M.R	M	20–23	No	No	No	No	No	No	Yes	Yes	No	Sometimes	25	Mild	No
E.R	M	27–30	No	No	No	No	No	No	No	Sometimes	Sometimes	No	10	Not present	No
E.C	M	20–23	No	No	No	No	No	No	No	Sometimes	No	Sometimes	10	Not present	No
M.S	M	20–23	No	No	No	No	No	No	No	No	No	No	0	Not present	No
S.P	F	24–26	No	No	No	No	Sometimes	No	Yes	Sometimes	No	Sometimes	25	Mild	No
GP.M	F	20–23	No	Sometimes	No	No	Sometimes	No	Yes	Yes	Yes	Yes	50	Moderate	Yes
G.R	F	24–26	Sometimes	Sometimes	No	No	No	No	Yes	No	No	Sometimes	25	Mild	No
E.V		24–26	No	No	No	No	No	Yes	Yes	Yes	No	Yes	40	Mild	No
L.P		20–23	No	No	No	Sometimes	Yes	No	No	No	No	Yes	25	Mild	No
L.V		27–30	No	No	Sometimes	No	No	No	Sometimes	Sometimes	No	Sometimes	20	Mild	No
S.T		24–26	Sometimes	Sometimes	Sometimes	Sometimes	Yes	Sometimes	Yes	Sometimes	No	Yes	60	Moderate	Yes
C.B		20–23	No	No	No	Yes	Yes	No	No	No	No	Yes	30	Mild	No
M.P		24–26	No	No	No	No	Sometimes	No	Yes	Yes	No	Sometimes	30	Mild	No
G.T		20–23	No	No	No	No	No	No	No	No	No	Sometimes	5	Not present	No
G.C		24–26	No	No	No	No	No	No	Yes	No	Yes	No	20	Mild	No
M.M		27–30	No	No	No	No	No	No	No	Sometimes	No	Sometimes	10	Mild	No
L.B		20–23	No	No	No	No	No	No	No	No	Yes	Yes	20	Mild	No
M.B		20–23	Sometimes	Sometimes	No	Sometimes	Sometimes	No	Yes	No	Yes	Yes	50	Moderate	Yes
V.R		24–26	Sometimes	Sometimes	Sometimes	No	Yes	No	Yes	Sometimes	Yes	Yes	60	Moderate	Yes
C.M		20–23	No	No	No	No	Sometimes	No	Sometimes	Yes	Yes	Yes	40	Mild	No
M.S		20–23	No	No	No	No	No	No	No	No	No	Yes	10	Not present	No
W.B		20–23	No	No	Sometimes	Sometimes	Sometimes	Sometimes	Yes	Sometimes	No	Sometimes	40	Mild	No
A.F		20–23	No	No	No	Sometimes	Sometimes	No	No	No	No	Sometimes	15	Not present	No
L.B		20–23	No	No	No	No	Sometimes	No	No	Yes	Yes	Sometimes	30	Mild	No
R.S		20–23	No	No	No	Sometimes	Sometimes	No	No	No	No	Sometimes	15	Not present	No
P.Z		27–30	Sometimes	Sometimes	No	Sometimes	No	Sometimes	No	No	Sometimes	Yes	35	Mild	No
M.L		20–23	Sometimes	Sometimes	No	Yes	Sometimes	Yes	Yes	Sometimes	Yes	Yes	70	Severe	Yes
AM.R		20–23	No	No	No	Yes	Yes	Sometimes	Yes	Yes	Yes	Yes	65	Moderate	Yes
V.B		24–26	No	No	No	Yes	Yes	No	No	Yes	No	Yes	40	Mild	No
ME.P		24–26	No	Sometimes	Sometimes	Yes	Sometimes	Sometimes	Yes	No	No	Yes	50	Moderate	Yes
